# Optimizing energy efficiency in wireless sensor networks through advanced cluster-based routing protocols

**DOI:** 10.1038/s41598-025-11382-3

**Published:** 2026-07-06

**Authors:** Fathia A. Mohamed, Emad S. Hassan, Moawad I. Dessouky, Basem El Elnaghi, Ahmed Magdy

**Affiliations:** 1 Department of Electronics and Electrical Communication, Faculty of Electronic Engineering, Menoufia University, Menouf, 32952 Egypt; 2 Department of Electrical Engineering, College of Engineering, Jazan University, 45142 Jizan, Saudi Arabia; 3 Electrical Engineering Department, Suez Canal University, Ismailia, Egypt

**Keywords:** WSNs, Clustering schemes, SEP, Cluster-heads election, Network’s lifetime, Throughput, Energy consumption, Engineering, Electrical and electronic engineering

## Abstract

Wireless sensor networks (WSNs) have gained significant attention in recent research due to their potential in various applications. However, energy conservation remains a critical challenge, as the nodes within these networks operate on non-replaceable batteries. This paper introduces a cluster-based proactive routing protocol designed for three-tier energy-heterogeneous WSNs, aiming to enhance network lifetime and energy efficiency. The proposed protocol incorporates a modified threshold-based cluster head (CH) selection mechanism, which considers both the energy levels and distances of sensor nodes (SNs) relative to the base station (BS). This approach prioritizes nodes with higher energy and closer proximity to the BS, increasing their likelihood of being elected as CHs. Furthermore, this protocol minimizes the formation of unnecessary CHs, particularly for SNs with low remaining energy, thereby reducing energy waste. Moreover, the proposed protocol is modified to address the challenge of long transmission distances for low-power nodes by strategically deploying them in specific network zones, thus further optimizing energy efficiency. Simulation results demonstrate that the modified protocol outperforms existing protocols such as SEP, IMPLEACH, and EEECA-THWSN in terms of total network lifetime, stability period (first node death), half-node death, last node death, throughput, and normalized remaining energy. Numerically, the proposed protocol with normal zone improves network performance, achieving an increase of up to 27% in network lifetime and throughput compared to other protocols.

## Introduction

Recently, wireless sensors have become a very important topic of research. Where, wireless sensor networks (WSNs) include of a few numbers of small, little-cost sensor nodes (SNs) that may be diffused randomly either statically or dynamically according to the desired network design^1^. Here, each node has a limited battery, its own sensing range and a communication port. All SNs wirelessly gather data from the surrounding environment to extract useful information^1–3^. Then they transmit all collected information data through the various techniques used for transmission to the base station (BS) or called sink^3,4^. Furthermore, SNs detect the physical properties or the environmental properties (like as; pressure, emissions, humidity and temperature as well) and then convert them into signals for monitoring, control, military and security purpose, agriculture, health monitoring, smart home and target tracking, etc^5,6^.

In WSN, the process of determining the path among both a source and a destination to send data is named routing^7^. There are many difficulties facing actual routing of SNs, the most important of which are the restricted energy of SNs, the network’s lifetime, how to deploy the SNs, the techniques used for sending information, as well as the distance related to SNs to the BS, etc^8–10^. Therefore, the main problem of the WSN resulting from the previous problems is the energy consumption associated with nodes in the network. Therefore, clustering technique is a well-researched approach to achieve energy-efficient balanced routing and reduce energy consumption, where it divides the SNs into clusters^9,11^. Every cluster can be headed by only one cluster head (CH) used for collecting data from SNs and finally sending it to the BS, in each cluster there are one or more non-CH node also named cluster members (CMs). Moreover, Clustering is an influential technique for minimizing the overheads of communication and reducing energy exhaustion. So that, we must be careful when selecting CH from live-SNs in the network as the remaining energy of the SN and the transmission distance must be considered^11,12^.

Here, clustering schemes have two types of clustering protocols called homogeneous-clustering schemes which are found in the homogeneous networks in which all SNs include the same type and initial power, likewise in the low energy adaptive-clustering hierarchy (LEACH)^13,14^. The heterogeneous-clustering schemes which are found in heterogeneous networks where SNs are different and contain different amounts of power and types of nodes^15–18^. Examples of these schemes are found in many protocols, for example: stable election protocol named; SEP^19^, enhanced-SEP named; ESEP^20^, Modified-SEP named; MSEP^21^, a prolong-SEP named; PSEP^22^, a zonal-SEP named; ZSEP^23^ also and in enhanced-ZSEP named; E-ZSEP^24^, etc. But one of the disadvantages of the homogeneous-cluster is that the instability period for nodes of the network is not good, unlike the heterogeneous-cluster, because after the first SN dies, the network breakdown because it contains the same level power and the same type of SN. While in the heterogeneous cluster there are different levels of power^13^.

In the homogeneous and heterogeneous networks, CH selection only depends on the initial energy of the live-SNs and doesn’t depend on an average energy related to the live-SNs. This may lead to the death of CH quickly, especially if its initial power is low and its long transmission distance to the BS.

This paper introduces an enhanced cluster-based proactive routing protocol designed for three-tier energy-heterogeneous WSNs, with the goal of improving energy efficiency and maintaining stable communication paths between SNs and the BS to extend the network’s lifetime. The proposed protocol incorporates a modified threshold formula that considers the energy levels and distances of all SNs relative to the BS, giving nodes with higher power and closer proximity a greater likelihood of being selected as CHs. Additionally, it reduces energy consumption during the cluster formation process by avoiding the creation of unnecessary CHs, particularly for SNs with low remaining energy, thereby minimizing energy waste within the network. However, the protocol faces challenges related to energy waste in normal nodes due to long transmission distances. To address this, a modified version of the protocol introduces a ‘normal zone’ (Nrm-Zone1), where normal nodes are strategically deployed to shorten their transmission distance to the BS, thereby reducing energy waste, enhancing energy efficiency, and improving the stability period, network lifetime, and throughput in WSNs.

## Related work

In WSNs, the goal of all SNs in the network is to sense the desired site field, gather all information and then transmit it to the BS. Initially, there are two transmission methods namely direct transmission (DT), and minimum energy transfer (MTE) used to send data to the BS in networks^25^. However, both techniques do not guarantee good distribution of the power load. This is because in the DT method, the SNs deliver their data directly to the BS, while in the minimum energy transfer (MTE), the data is transmitted over a path with the lowest possible cost. Thus, the clustering technique leads to equal distribution of the power load in WSN, reduces energy exhaustion and also increases the network’s lifetime^12^.

LEACH^14^ is perhaps the first and most important clustering-routing protocol of homogeneous WSN (all types of SNs in the network have an equal amount of energy). Also, it uses an adaptive clustering scheme. Where it creates clusters, in each cluster it elects CHs used to collect data from member node and deliver it to the BS after aggregating. The operation process in LEACH consists of multiple rounds in which every round is split into the two phases namely, the set-up and the steady-state phase. During setup phase: the formation of clusters is done, and CHs are elected, while during the steady-state phase: sensed data related to SN is combined and transmitted to the BS.

Each node will take a chance for being a CH exactly once in every (*1/p*_*i*_) round. Randomly, every SN creates its random number (RN) that lies between 0 and 1. The node will take a chance to be a CH during the current round only if RN has value less than the actual threshold value *T(n*_*i*_*)*. therefore, the desired formula of the threshold in LEACH is estimated as:

1 $$T\left( {n_{i} } \right) = \left\{ {\begin{array}{*{20}l} {\frac{{p_{i} }}{{1 - p_{i} \left( {r \times mod\frac{1}{{p_{i} }}} \right)}}} \hfill & {if\;n_{i} \in G} \hfill \\ {0,} \hfill & {Otherwise} \hfill \\ \end{array} } \right.$$

Here, *r*_*i*_ denotes the current round, *P*_*i*_ is the probability that means the required percentage of all SNs to be CHs, and *G* denotes the set of all SNs won’t be CHs in the last 1/*p*_*i*_ rounds. Where, the node which was elected as CH will not be chosen again in the next successful 1/*p*_*i*_ rounds. The disadvantages of LEACH protocol are that it cannot consider the factors of energy and distance of nodes for the election of CHs.

Therefore, there are many advanced protocols for LEACH, these protocols are extension of LEACH such as: LEACH deterministic CH Selection (LEACH-DCHS)^26^, LEACH with distance-dependent threshold (LEACH-DT)^27^ and distance and energy aware-LEACH (DE-LEACH)^28^. In LEACH-DCHS, the CHs selection depends on the residual power level of the node. On the other hand, the distances between sensors and the base station in LEACH-DT are taken into consideration during the CH election to achieve the balanced energy consumption between sensors. Furthermore, DE-LEACH elects CHs depending on the factor of distance and residual power of the nodes, where this protocol takes both the shorts distance factor and energy factor in the formula of the threshold containing multi-hop routing. But in the heterogeneity of sensor nodes, the LEACH protocol the optimal choice of CH is not guaranteed. However, LEACH works very well for homogeneous networks, but it isn’t suitable for heterogeneous networks. So that, many various protocols are presented of the heterogeneous networks as presented next.

In^19^, SEP for heterogeneous WSN that has two-levels power of SNs, where it guarantees network’s reliability. SEP uses a clustering technique similar to that used for the LEACH protocol for selecting CHs according to their initial or starting energy, and this selection is based on weighted election probability. Moreover, this protocol has a different classification of SNs used in the network than used in LEACH. In SEP, all SNs are categorized into (advanced and normal nodes). Clearly, the advanced nodes contain higher power than the energy used for normal nodes. So that, advanced nodes contain more chance to be CHs due to their high energy level. Therefore, this protocol achieves greater network’s lifetime, stability period for network, and throughput.

In^20^, ESEP is the stretching of protocol SEP, uses a clustering technique like that used in the SEP protocol for selecting CHs from SNs. It achieves better energy-efficiency than SEP as it contains three heterogeneous levels of energy in which the SNs are categorized into (advanced, intermediate and normal nodes). Modified-SEP (MSEP)^21^ contains two types of nodes as in SEP, but it differs from SEP in the CH selection criteria, as it depends on the remaining power associated with each SN and the average network power. However, it is unable to keep the network’s efficiency in terms of lifetime.

In^29^, the efficient MSEP (EM-SEP) distributes the power evenly among the nodes in the clusters to enhance network’s stability. So, there are CHs at any given time. But this actually results in higher energy consumption per round. As EM-SEP is not able to decrease the power utilizing of network. On the other hand, a PSEP^22^ can improve the network’s lifetime where, it utilizes the average remaining energy in threshold value to maintain balanced energy consumption.

In^23^, the ZSEP is also stretching of SEP, has two levels of energy. It depends on the network field division into three-zones: (zone 0) for normal nodes, (head zone 1) and (head zone 2) for advanced nodes. Also, it uses two methods for transmission: the transmission via CHs for higher SNs energy and direct transmission for lower SNs energy to BS. But the SNs with small power die quickly in DT. Therefore, EZSEP is presented in^24^ to solve the problem of any remote SN in ZSEP, follows the same technique used in ZSEP, besides dividing the network domain into specific areas. In addition to, the Threshold-based EZSEP (TE‐ZSEP) improves the network’s lifetime and throughput than in EZSEP by redefining the threshold-formula in EZSEP based on both weighted-energy and distance parameters^24^.

In^30^, improved ZSEP (IZ-SEP) contains two levels of power as ZSEP and two zones. It extends the network’s lifetime by decreasing the power dissipation. In which every CH would be selected from SNs depending on the residual energy respected to every SN as well as the number of neighbors of each node during the cluster. Moreover, in^31^ a distributed energy-efficient clustering protocol known as the (DEEC) procedure with different power levels, where it selects the CH from nodes depending on both the initial and residual power.

In^32^, the approach called Distance Based SEP (DB-SEP) achieves better energy efficiency and network’s lifetime comparing SEP protocol, it contains two levels of power and based on both of their initial or start energy as well as their short distances between them and the BS.

Clearly, the three level (Tire) heterogeneous SNs were diffused for studying the lifetime, throughput and stability period of the network. In^33^, TSEP has three types of nodes as ESEP with various power values. Also, this protocol is called a threshold-sensitive protocol depending on two thresholds named (hard and soft threshold), and CHs election depends on the threshold, so it can’t keep the energy-efficiently for guaranteeing the balanced distribution of load.

In^34^, enhance threshold-sensitive SEP which is known as ET-SSEP modifies TSEP. The actual CH in the network was elected based on their remaining power level and the actual minimum number of clusters in each round. Clearly, in^35^, the residual power and location associated with nodes have a strong function in the CH election process to improve clustering protocol as applied in the Improved LEACH protocol (Imp-LEACH). The Distance based Enhance-TSEP (DE-TSSEP) Protocol was presented in^36^. In this approach, the actual CH in the network depended on nodes’ remaining energy, networks average energy as well as the short distance among CH and the BS.

In^37^, distance-aware and energy-efficient residual-SEP (DARE-SEP) contains the three heterogeneous powers or levels and is the hybrid approach. In DARE-SEP, the CHs were elected on the basis of the residual energy of SNs and the closer distance among CHs and BS. But in this approach, the selections of actual SNs that should be CHs are not verified. In^38^ enhanced energy efficient clustering approach for three-tier heterogeneous WSNs (EEECA-THWSN), each SN chooses itself as cluster head according to its own threshold-function which depends on the energy and distance of SNs in each round from the BS to the SNs. But this approach does not depend on the average energy of a live-SNs selected as CH, so a number of unnecessary clusters appear, causing power loss in the network.

The previously mentioned protocols select CHs based solely on an absolute threshold value, without taking into account the average energy of the live SNs chosen as CHs. This approach can lead to the formation of an excessive number of CHs, which often deplete their energy quickly, especially if they have low remaining energy and are located far from the BS. Consequently, significant energy is wasted during the clustering process due to the rapid energy consumption of these CHs. Therefore, this paper proposes a proactive routing protocol designed for three-tier energy-heterogeneous networks, aiming to maximize energy efficiency, minimize energy waste, and maintain reliable communication paths between SNs and the BS to extend the network’s lifetime. The key contributions of this paper are summarized as follows:

1. *Enhanced CH Selection Protocol* We propose a protocol that reduces energy waste by modifying the weighted probability selection formulas. Unlike previous protocols that rely solely on the initial energy of SNs, our approach incorporates both the average energy of live SNs and the initial energy. This adjustment prevents the formation of unnecessary CHs, particularly for SNs with low remaining energy. Additionally, the actual threshold value is refined by considering both energy and distance factors.
2. *Improved Energy Consumption Protocol* A modified protocol, building on the first, further enhances energy efficiency by minimizing the transmission distance for low-power nodes (normal nodes) relative to the BS. This is achieved by strategically placing these nodes within a designated area (Nrm-Zone1), thereby reducing the premature death of cluster heads and extending the stability period.
3. *Performance Gains* The proposed protocol demonstrates significant improvements in various performance metrics, including the stability period (time until the first node dies), overall network lifetime, data packet delivery to CHs, energy consumption, and throughput, outperforming existing protocols such as SEP, IMP-LEACHM, and EEECA-THWSN.

The remainder of this paper is organized as follows: “System models” section discusses the system models. “The proposed protocol” section outlines the operation of the proposed protocols. “Network models of the proposed protocol” section details the network model for the proposed protocols. “Simulation results and analysis” section presents the simulation results and analysis. Finally, “Conclusion” section concludes the paper.

## System models

This section covers various models used to implement the proposed routing protocol, including the energy model and the energy dissipation model. A brief overview of each model is provided below.

### Energy dissipation model

**Fig. 1:**
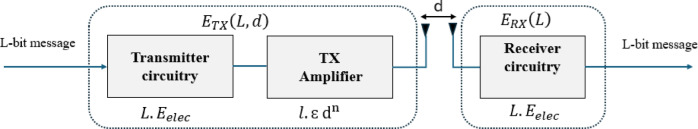
Energy dissipation model.

The basic operations in WSNs are data transmission and reception. Conventionally, the process of transmitting data in the network consumes a lot of energy during transmission, unlike receiving data^2^. In which, the life of a wireless SN builds on the lifetime of its battery. In this model, the radio energy dissipation model is clarified as in Fig. 1. Where, the energy consumed by transmitting an *L*-bit message through *d* which is a certain distance, is calculated as in Eq. (2):

2 $$E_{TX} \left( {L,d} \right) = \left\{ {\begin{array}{*{20}l} {L \times \left( {E_{elec} + \varepsilon_{fs } d^{2} } \right)} \hfill & {if\;d < d_{o} } \hfill \\ {L \times \left( {E_{elec} + \varepsilon_{amp } d^{4} } \right)} \hfill & {if\;d \ge d_{o} } \hfill \\ \end{array} } \right.$$

where *E*_*elec*_ represents energy dissipated per bit to turn on both transmitter electronic circuit (TX) and receiver electronic (RX) circuit that relates to a lot of parameters as (filtering, spreading, digital coding, and modulation of signal).

Clearly, ε_*fs*_ and ε_*amp*_ separately represent the amplification parameters of both transmitter to the free space and the multipath fading-models. In which the distance among (TX) and (RX) is denoted by *d* and the threshold distance is *d*_*o*_ calculated as:

3 $$d_{o} = \sqrt {\frac{{\varepsilon_{fs} }}{{\varepsilon_{amp} }}}$$

Also, an energy expanded for receiving an L − bit message the radio expends can be given as:

4 $${E}_{RX(l)}=L\times {E}_{elec}$$

### Energy model

Clearly, the important criteria in the process of designing every routing-protocol in WSNs are energy efficiency and energy balancing. Then the heterogeneity in WSN varies to computational heterogeneity energy-heterogeneity (due to various types of nodes with different power level) and link heterogeneity (due to different transmission techniques among them). In this paper, we can introduce an advanced technique for routing data in a heterogeneous network to monitor SNs energy wasting. Moreover, the three-tire heterogeneity with respect to initial or start node energy can be considered. So, we will address the three levels of power-heterogeneity named: (advanced node, intermediate and normal node). Where the intermediate nodes’ initial energy is higher than an initial energy of normal nodes and lower than an initial energy of advanced nodes. Assume that *n*_*i*_ is called the total SNs in the network. We assume that *b*, *m* are the proportions of intermediate and advanced nodes separately. Clearly, the energy level of intermediate nodes is *µ* $\upmu$ $\upmu$ times more than the energy level of normal nodes and the energy level advanced nodes is *α* times more than the energy level of normal nodes and, where *μ* = *α/2*. So that the initial energy of each node called (normal, intermediate and advanced node) *E*_*nrm*_, *E*_*int*_ and *E*_*adv*_ respectively, will be introduced in Eqs. (5, 6 and 7):

5 $${E}_{nrm}={E}_{o}$$

6 $${E}_{int}={E}_{o }\left(1+\mu \right)$$

7 $${E}_{adv}={E}_{o }\left(1+\alpha \right)$$

Therefore, the total initial energy (*E*_*total int*_) used in the system will be expressed as in Eq. (8)

8 $$\begin{aligned} E_{total int} = & n_{i} E_{o} \left( {1 - m - b} \right) + n_{i} mE_{o} \left( {1 + \alpha } \right) + n_{i} bE_{o} \left( {1 + \mu } \right) \\ & = n_{i} E_{o} \left( {1 + \alpha m + b\mu } \right) \\ \end{aligned}$$

## The proposed protocol

In this section, we introduce the enhanced cluster-based proactive routing protocol for three-tier energy-heterogeneous WSNs, designed to increase the stability period (FDN), extend network lifetime, reduce energy waste from long transmission distances, and improve overall energy consumption. The details of this protocol are explained as follows:

The proposed protocol has three heterogeneous-power levels as mentioned above (advanced, intermediate and normal nodes) randomly spread in the network domain. The main purpose is to expand the network’s lifetime from the network starting its operation to the last dead node and reducing power consumption. As, all nodes don’t have the same residual energy therefore, this proposal aims to reduce CH extinction from low-energy nodes because each node gets only one chance to become a CH. According to CH selection process, this proposal doesn’t depend only on the initial energy in the selecting of CHs as mentioned in LEACH, SEP, Imp-LEACH and EEECA-THWSN, but it considers both the initial energy of each SN in the network and the average energy of a live-SNs. Furthermore, CHs are determined by adjusting the actual threshold *T(n*_*i*_*)* in order to identify the suitable nodes to become CHs. Due to these motivations, we also adjust the formula of threshold value based on both the energy and the distance ratio between both sensor nodes and BS also this value relates with the new formula of waited election probabilities defined in Eqs. (9, 10 and 11) depended on the average energy of a live-SNs to select actual CHs. As a result, the chance of low-power nodes becoming CHs is reduced, resulting in an improvement in the network stability period. This is explained as follows:

- Weighted Election Probabilities for Nodes

Let, *P*_*i*_ is the probability of all SNs (*n*_*i*_*)* to be selection-CHs for *r*_*i*_ the number of rounds, it varies according to the types of nodes called (advanced, intermediate and normal nodes). *P*_*i*_(*nrm*), *P*_*i*_(*int*) and *P*_*i*_(*adv*) represent respectively weighted probabilities of normal, intermediate and advanced nodes. These values of weight probabilities based on the initial (start) energy (*E*_*o*_) of SNs also and the average energy of a live-SNs *E*_*avg*_(*r*_*i*_). So that, the modified formula of CH election probabilities related with normal, intermediate and advanced nodes are ${{P}_{i}}_{(nrm)}$, ${{P}_{i}}_{(int)}$ and ${{P}_{i}}_{(adv)}$ given in Eqs. (9, 10 and11), respectively.

9 $${{P}_{i}}_{(nrm)}=\frac{{P}_{i}*{{E}_{avg}(r}_{i})}{\left(1+\alpha m+b\mu \right)*{E}_{o}}$$

10 $${{P}_{i}}_{(int)}=\frac{{P}_{i}\left(1+\mu \right)*{{E}_{avg}(r}_{i})}{\left(1+\alpha m+b\mu \right)*{E}_{o}}$$

11 $${{P}_{i}}_{(adv)}=\frac{{P}_{i}{{\left(1+a\right)*E}_{avg}(r}_{i})}{\left(1+\alpha m+b\mu \right)*{E}_{o}}$$

In which* E*_*avg*_(*r*_*i*_) considers the average energy of a live-SNs shown in Eq. (12)

12 $$E_{avg} (r_{i} ) = \frac{1}{n}\mathop \sum \limits_{j = 1}^{n} E_{nj} \left( {r_{i} } \right),\;if\;E_{nj} \left( {r_{i} } \right) > 0$$

where, *E*_*nj*_(*r*_*i*_) represents the energy of SN *n*_*j*_ per round *r*_*i*_ and *n* represents the total number of a live-SNs that like *n*_*i*_ before the first death of SN.

Generally, each node will become a CH with the actual probability *P*_*i*_ where, each node only gets one chance to become a CH every (1/ *P*_*i*_) rounds. So (*n*_*j*_ × *P*_*i*_) is the average number of the CHs.

(b) Threshold concepts for CHs selection

Since the decision of SNs of being a CHs depends on the value of threshold of SN as mentioned in LEACH, SEP, Imp-LEACH and EEECA-THWSN. So that, we modify threshold formula mentioned in^38^ based on both the modified formula of CH election probabilities, the ratio of the current energy of the nodes (*E*_*current*_) and the initial energy of the nodes (*E*_*initial*_) as well as the ratio of the distance from the stationary BS of SNs (*D*_*current*_), at the current round, and the highest distance between SNs to the stationary BS (*D*_*max*_). In which, the distance between the stationary BS and the SNs must become short. Besides, the distance from the sensor nodes to the base station (d_BS_) is also added in the modified threshold formula designed for CH selection. Here, each node can produce a random number. This actual value can locate between (0 and 1). The new modified formula for the threshold is set as in Eqs. (13, 14 and 15). The values of *T*(*n*_*i*_)_*nrm*_, *T*(*n*_*i*_)_*int*_, and *T*(*n*_*i*_)_*adv*_, respectively, for normal, intermediate and advanced nodes are compared with the actual threshold value generated randomly. Here, if these threshold values are obtained larger than the randomly generated values, the sensors nodes should be CHs.

13 $$T(n_{i} )_{nrm} = \left\{ {\begin{array}{*{20}l} {\frac{{P_{{iH\left( {nrm} \right)}} *\left( {w\left( {\frac{{ E_{current} }}{{ E_{initial} }}} \right) + x\left( {\frac{{ D_{current} }}{{ D_{\max } }}} \right) + \left( {\frac{1}{{ d_{Bs} }}} \right)} \right)}}{{1 - P_{{i\left( {nrm} \right)}} \left( {r \times \bmod \frac{1}{{P_{{i\left( {nrm} \right)}} }}} \right)}}} \hfill & {if n_{{i\left( {nrm} \right)}} \in G} \hfill \\ {0,} \hfill & {Otherwise} \hfill \\ \end{array} } \right.$$

14 $$T(n_{i} )_{int} = \left\{ {\begin{array}{*{20}l} {\frac{{P_{{iH\left( {int} \right)}} * \left( {{\mathrm{w}}\left( {\frac{{ E_{current} }}{{ E_{initial} }}} \right) + {\mathrm{x}}\left( {\frac{{ D_{current} }}{{ D_{max} }}} \right) + \left( {\frac{1}{{ d_{BS} }}} \right)} \right)}}{{1 - P_{{i\left( {int} \right)}} \left( {r \times mod\frac{1}{{P_{{i\left( {int} \right)}} }}} \right)}}} \hfill & {if\; n_{{i\left( {int} \right)}} \in G^{\prime \prime } } \hfill \\ {0,} \hfill & {otherwise} \hfill \\ \end{array} } \right.$$

15 $$T(n_{i} )_{adv} = \left\{ {\begin{array}{*{20}l} {\frac{{P_{{iH\left( {adv} \right)}} * \left( {w\left( {\frac{{ E_{current} }}{{ E_{initial} }}} \right) + x\left( {\frac{{ D_{current} }}{{ D_{\max } }}} \right) + \left( {\frac{1}{{ d_{BS} }}} \right)} \right)}}{{1 - P_{{i\left( {adv} \right)}} \left( {r \times \bmod \frac{1}{{P_{{i\left( {adv} \right)}} }}} \right)}}} \hfill & { if\;n_{{i\left( {adv} \right)}} \in G^{\prime \prime \prime } } \hfill \\ {0,} \hfill & {Otherwise} \hfill \\ \end{array} } \right.$$

In which, ${{P}_{iH}}_{(nrm)}=H*{{P}_{i}}_{(nrm)}$, ${{P}_{iH}}_{(int)}=H*{{P}_{i}}_{(int)}$ and ${{P}_{iH}}_{(adv)}=H*{{P}_{i}}_{(adv)}$ represent separately weighted probabilities for both normal, intermediate and advanced nodes. *H* will become the proportional constraint depending on the size of network, with a value ranging from 0 to 1. Furthermore, *G'*, *G''*, *G'''* separately represent the set or group of both the normal, intermediate and advanced nodes. These types of SNs will not become selection-CHs within the last ${1/{P}_{i}}_{(nrm)}$, ${1/{P}_{i}}_{(int)}$ and ${1/{P}_{i}}_{(adv)}$ rounds, respectively. Where *w* and *x* are the ratio factors whose values lie between 0 and 1; (0 < *w* < 1), (0 < *x* < 1) and *w* + *x* = 1.

In which (*x*_*i*_, *y*_*i*_) is the coordinate of the SNs (*n*_*i*_) and the coordinate of the stationary BS is (*x*_*BS*_, *y*_*BS*_). So, the distance among BS and any member node ${d}_{i to BS}$ in the network will be as in in Eq. (16)

16 $${d}_{i to BS}=\sqrt{{{(x}_{BS-}{x}_{i})}^{2}+{({(\mathrm{y}}_{BS-}{\mathrm{y}}_{i})}^{2}}$$

Furthermore, the coordinates of CHs can be as (*x*_*CH*_, *y*_*CH*_), so the distance between both the base station and the member CHs ${d}_{CH to BS}$ will be estimated as in Eq. (17)

17 $${d}_{CH to BS}=\sqrt{{{(x}_{BS-}{x}_{CH})}^{2}+{({(\mathrm{y}}_{BS-}{\mathrm{y}}_{CH})}^{2}}$$

To address the issue of long-distance transmission for low-power nodes (normal nodes) in the proposed protocol, we recognize that sensor nodes or cluster heads, particularly those with lower energy, may die quickly due to the large transmission distance to the stationary base station (BS). Normal nodes have less initial energy (*E*_*o*_) compared to intermediate and advanced nodes, making them more vulnerable. To improve network performance and reduce energy waste, we propose a modified version of the protocol. In this modified protocol, normal nodes are deployed in a designated area called Nrm-Zone1, while advanced and intermediate nodes are distributed across the same network domain as in the original protocol without a normal zone. This approach reduces the premature death of cluster heads, particularly those from normal nodes, and enhances the stability period (FDN) and overall network lifetime more effectively than the original protocol. The total network area is divided into two zones: Nrm-Zone1 and Zone 0, as described below:

- *Nrm-Zone1* This is the first zone, where normal nodes are distributed.
- *Zone 0* This is the second zone, encompassing the entire network domain without further division, where intermediate and advanced nodes are distributed.

**Fig. 2:**
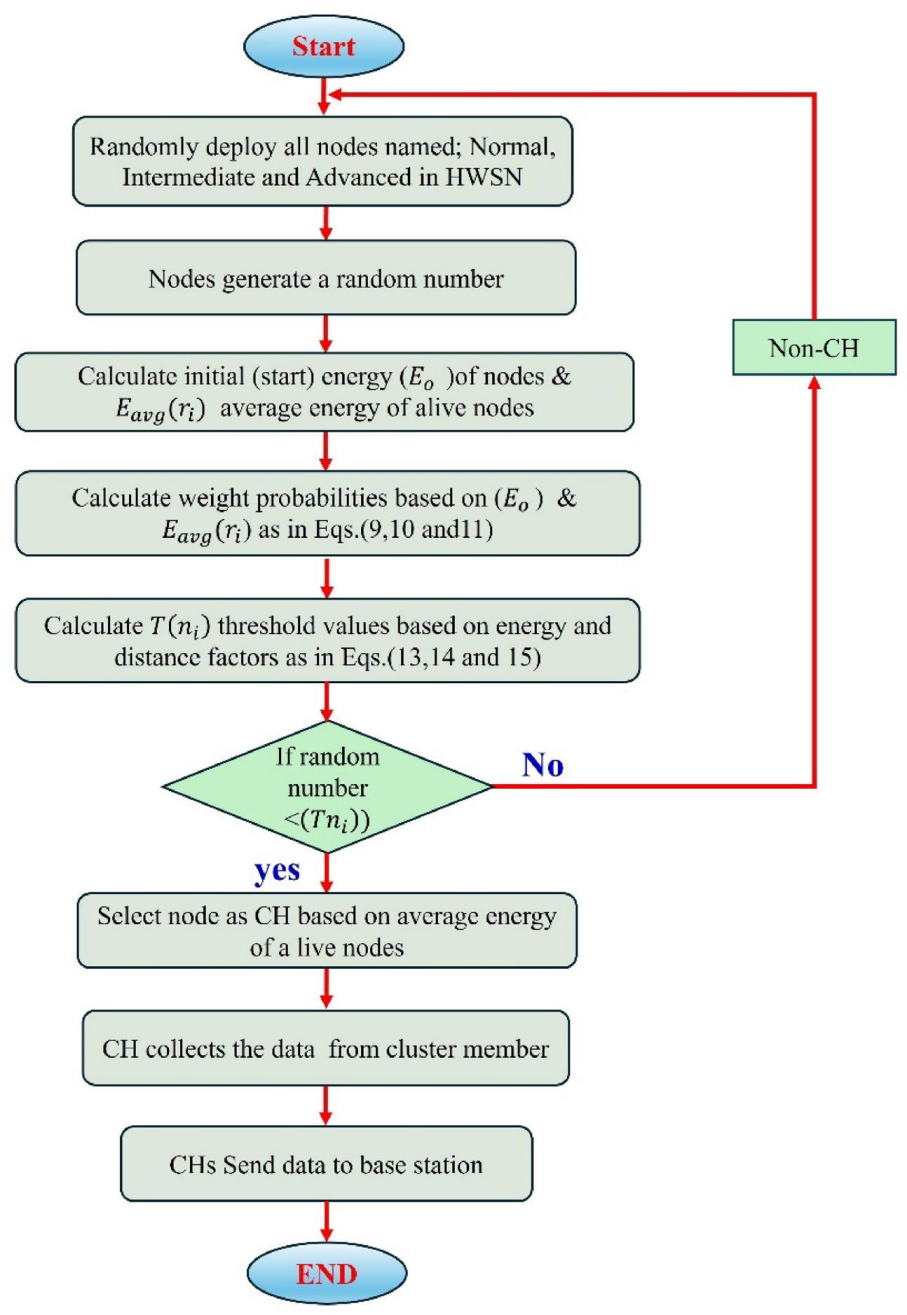
The flow chart of the proposed protocol.

As previously mentioned, the weighted selection probabilities are calculated using the same scenarios outlined in Eqs. (9, 10, and 11), while the threshold values for normal, intermediate, and advanced nodes are determined as shown in Eqs. (13, 14, and 15). Figure 2 illustrates the flowchart of the proposed protocol, and the corresponding protocol is presented in Table 1.

**Table 1:**
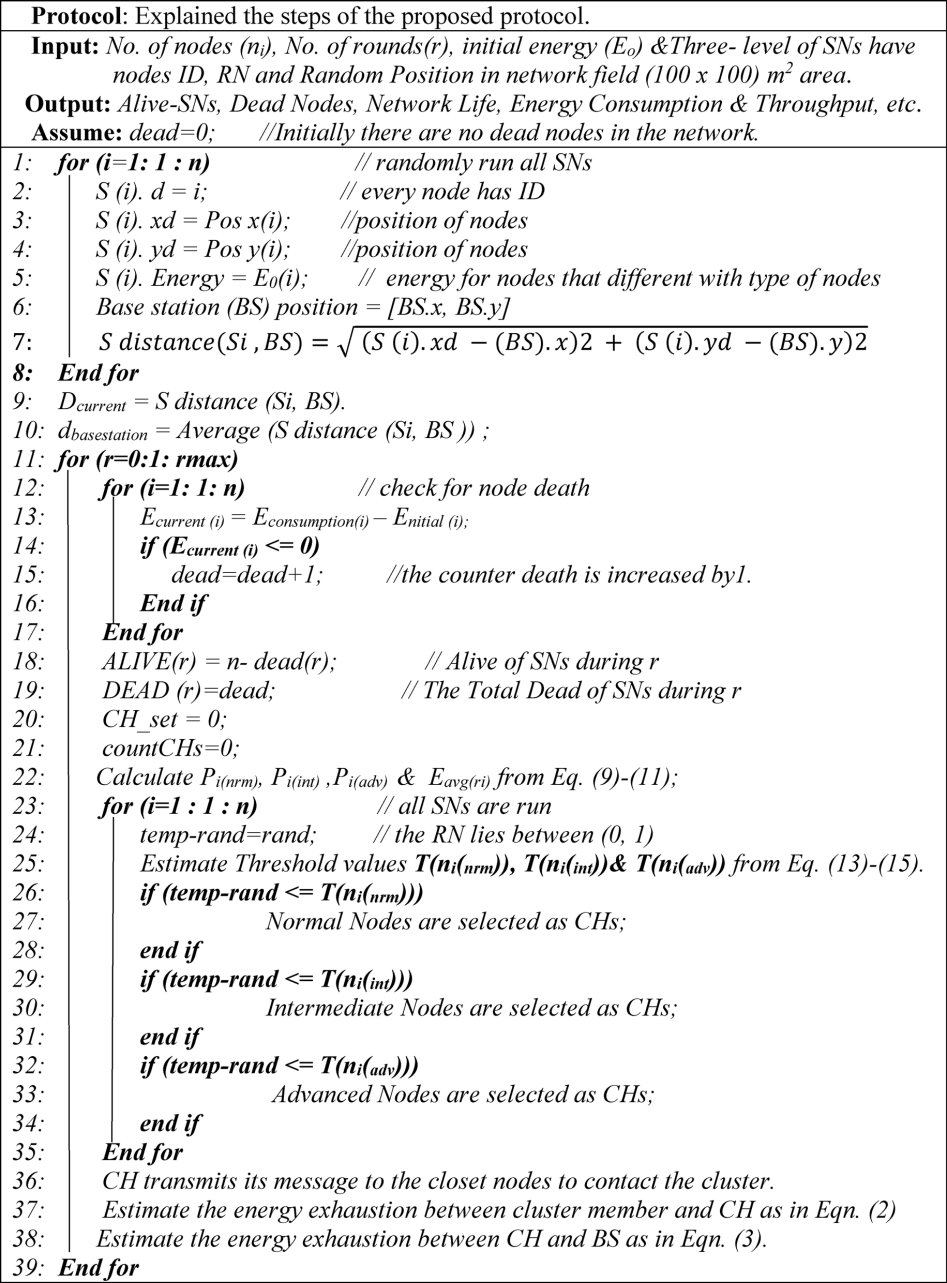
The proposed protocol steps.

## Network models of the proposed protocol

**Fig. 3:**
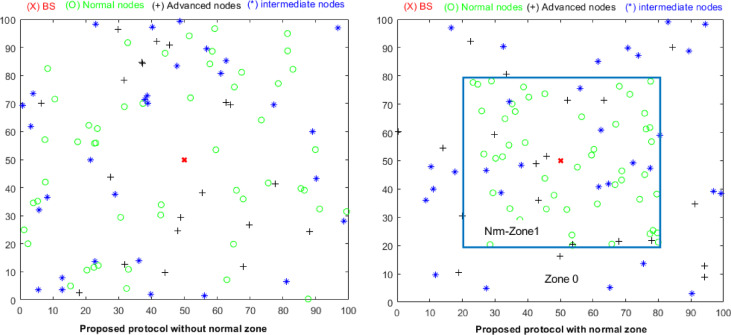
The network architecture models for (**a**) the proposed protocol without normal zone and (**b**) the proposed protocol without normal zone: 50% of normal SN in (Nrm-Zone1).

The network models for the proposed protocol and the modified protocol with normal zone will be explained in this section. Also how to diffuse the SNs in the field area is also presented. We assume the network field is a square whose area is A = M × M, where M = 100 m, SNs in the network equal to 100 heterogeneous SNs are randomly published and in the middle of the network there is a base station (BS). Clearly, we show the network models of proposed protocol as in Fig. 3, where it is explained in detail as follows:

In the proposed protocol without normal zone, 100% of the SNs named (normal, intermedia and advanced nodes) are randomly distributed in the network field in the area A = [100 × 100 m^2^].

In the modified protocol with normal zone where the SNs nodes are deployed randomly as follow:

- Nrm-Zone1: 50% of the nodes are considered the normal nodes diffused randomly in the following coordinates (20 ≤ X ≤ 80), (20 ≤ Y ≤ 80).
- Zone 0: contains 20% of the advanced nodes and 30% of the intermediate nodes are diffused in the network field in the square meter (100 × 100), bounded by (100 ≤ X ≤ 100), (100 ≤ Y ≤ 100).

## Simulation results and analysis

### Simulation setting

The simulation is performed in MATLAB depending on the simulation model in which the total number of sensor nodes used is 100 heterogeneous SNs randomly distributed in the network field with an area of 100 × 100 square meters. Moreover, the location of the stationary BS is in the middle of the sensing environment and the energy used for all nodes is fixed for all protocols, which can become 80 Joules. The simulation parameters used to evaluate the proposed protocol during the simulation model are listed in Table 2. We present the performance analysis of the proposed protocol in comparison with existing routing protocols, including SEP, Imp-LEACH, and EEECA-THWSN. Additionally, the performance metrics used to evaluate the proposed protocol are explained as follows:

- Dead Nodes are the total number of SNs whose energy is less than or equal to zero, counted as the dead nodes during each round.
- Packets to CHs represents the number of packets that will be delivered to CHs.
- Throughput named Packet to BS represents the number of packets that will be transmit from CHs of SNs to the stationary BS.
- Stability period: represents the no. of rounds measured when the network begins its operating until the first death.
- Instability period: represents the no. of rounds measured at the first death and the last death of SN.
- Network lifetime: represents the no. of rounds measured when the network start operating until the last node in the network dies (LND). Therefore, three metrics are calculated namely, first node dies (FND), half nodes die (HND), as well as last node dies (LND).

**Table 2:** The simulation parameters.

Parameter	Value
Network area	100 × 100 m^2^
r_i_ (No. of round)	8000 rounds
n_i_ (No. of SNs)	*100* SNs
*E*_*o*_ = *E*_*nrm*_(initial energy of Normal SNs )	0.5 J
*E*_*adv*_ (initial energy of advanced SNs)	*E*_*o*_ (1 + *α*)
*E*_*int*_ (initial energy of Intermediate SNs)	*E*_*o*_ (1 + *μ*)
*E*_*elec*_ (Tx/Rx electronic energy)	50 nJ/bit
$\varepsilon$_*fs*_ (Energy dissipation for free space models)	10 nJ/bit/m^2^
$\varepsilon$_amp_ (Energy dissipation for multipath fading models)	0.0013 pJ/bit/m^4^
*E*_*DA*_ (Energy for data-aggregation)	5 nJ/bit/signal
*P*_*i*_ (The optimal probability)	0.1
*L* (Packet transmitted size)	4000 bits

The simulation results of the proposed protocol are tailored for a heterogeneous network with three tiers of SNs, where we assume m = 0.2 and α = 1.71428. It is evident that the advanced nodes, totaling 20 SNs, have an additional energy level of α = 1.71428 compared to the normal nodes. Additionally, with b = 0.3 and μ = 0.85714, the number of intermediate nodes is 30 SNs, with an extra energy level of μ = α/2 = 0.85714 above that of the normal nodes.

**Fig. 4:**
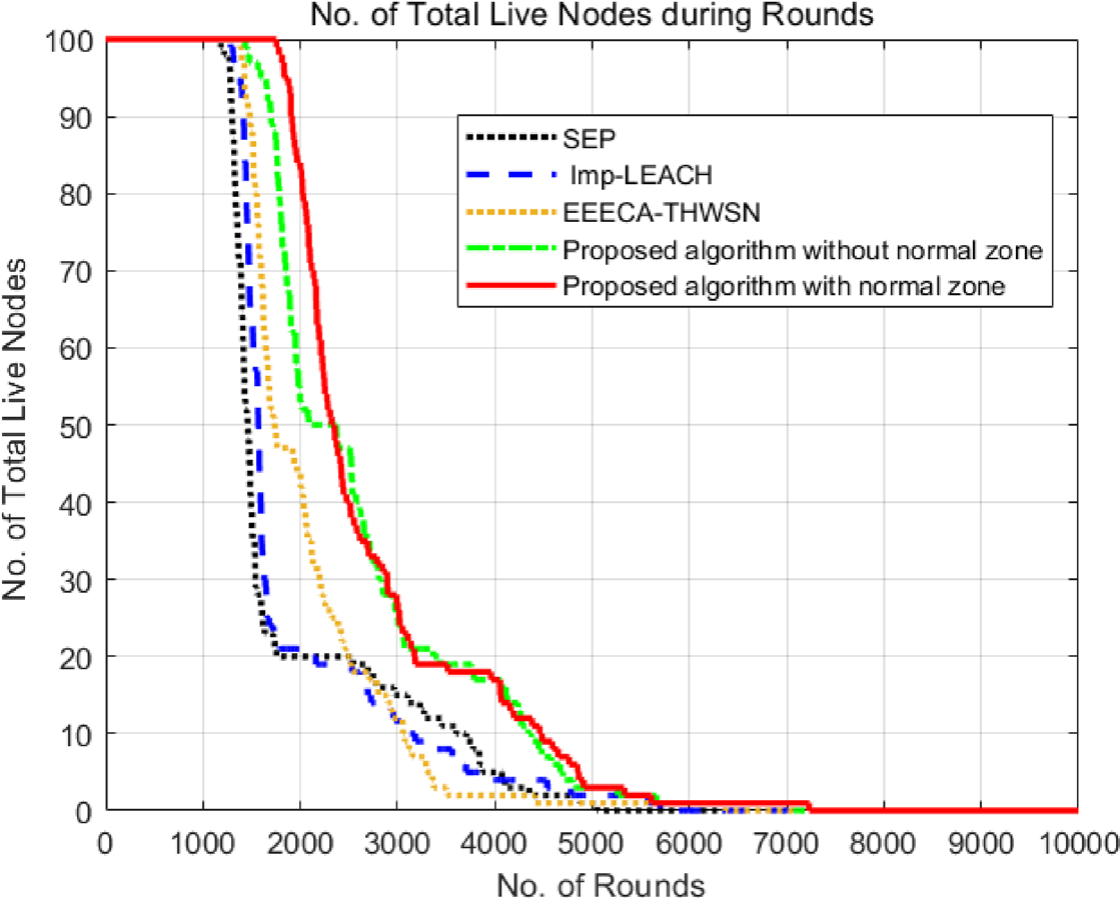
Total number of live nodes versus number of rounds for the proposed protocols with and without the normal zone and SEP, Imp-LEACH, and EEECA-THWSN.

**Fig. 5:**
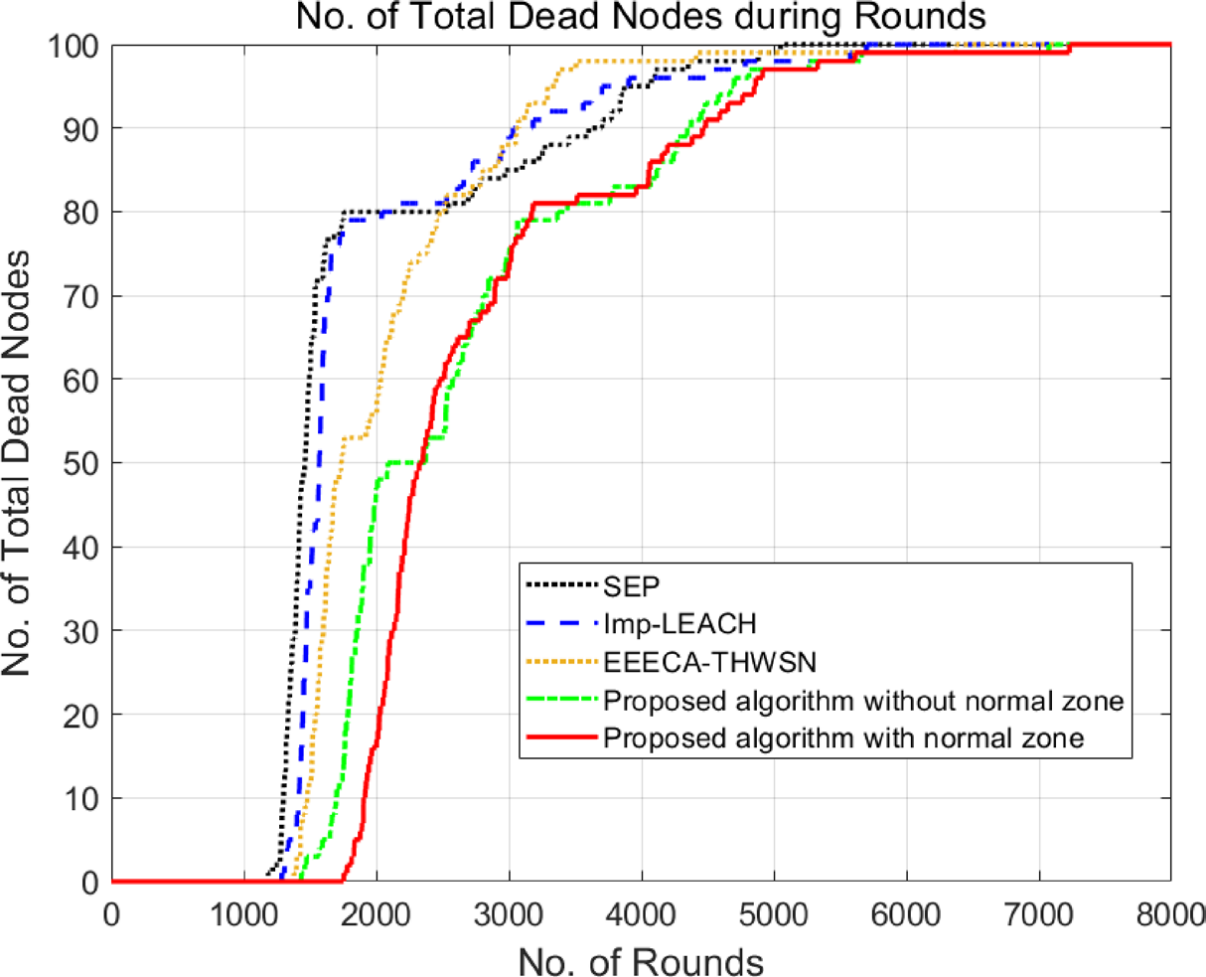
The total number of dead nodes versus number of rounds, the first dead node (FDN) of the proposed protocol with the normal zone occurs at round 1755, and the LDN at round 7235 than SEP, Imp-LEACH, and EEECA-THWSN.

**Fig. 6:**
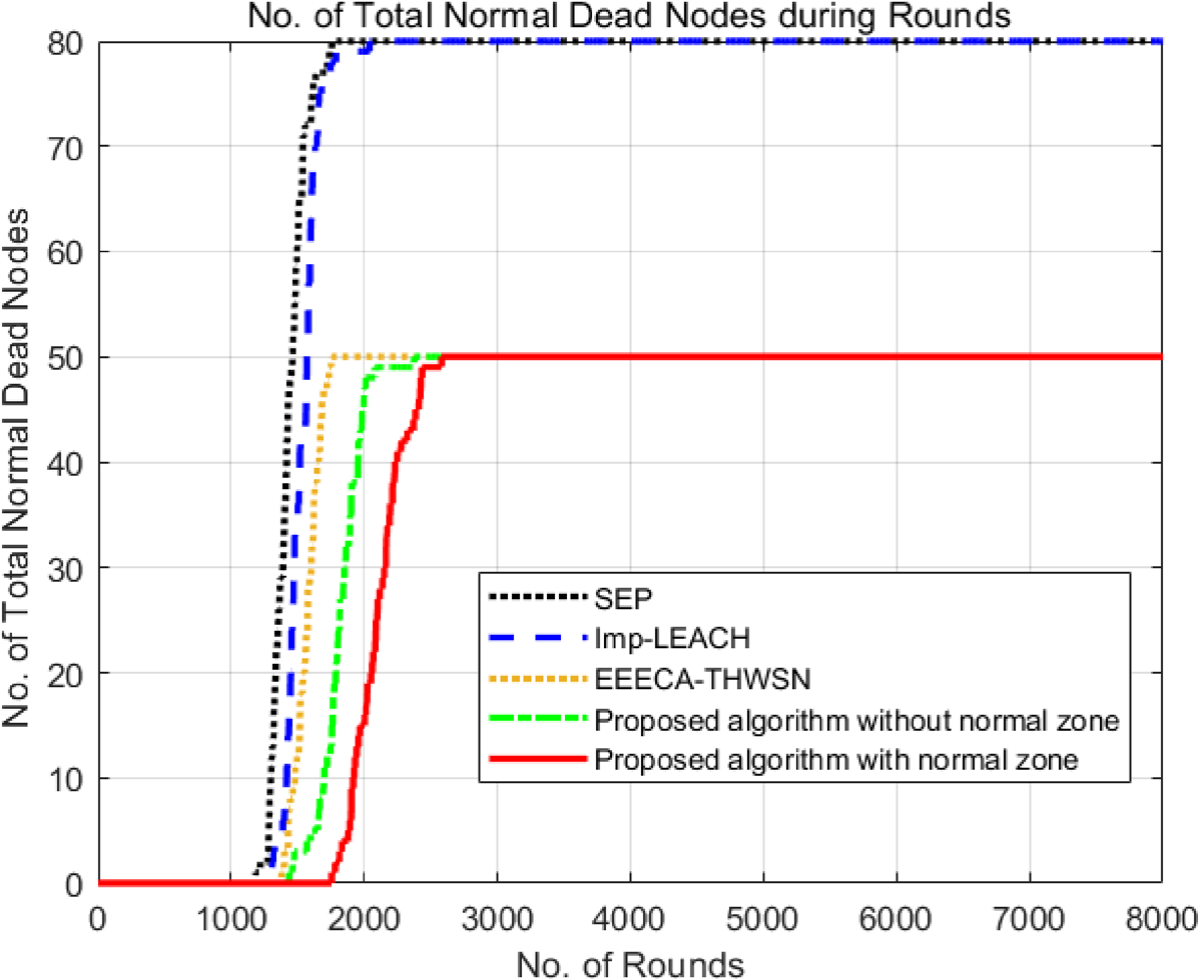
The total number of normal dead nodes against number of rounds, the FDN of normal nodes at the proposed protocol with the normal zone occurs at round 1755.

**Fig. 7:**
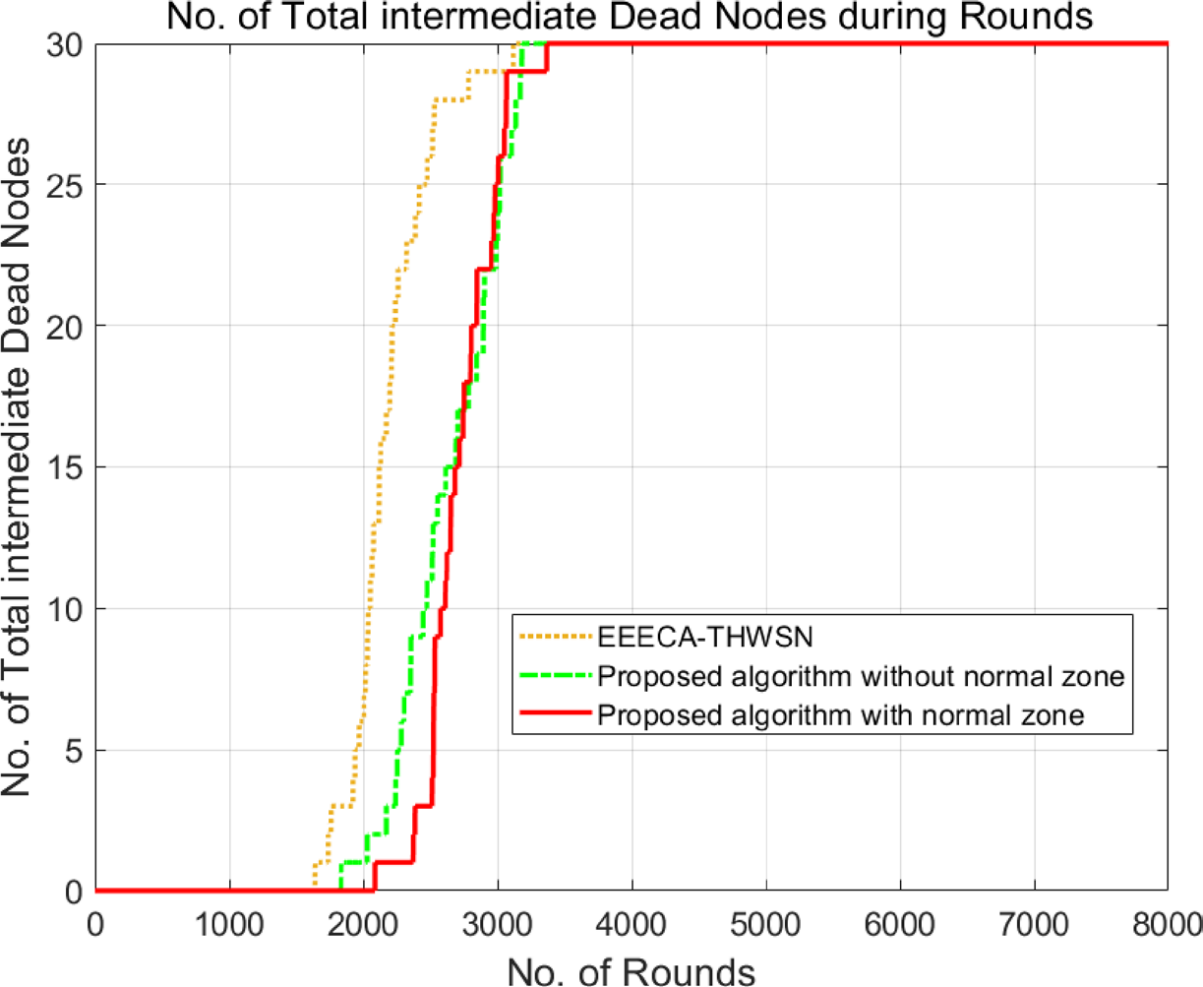
The total number of intermediate dead nodes against number of rounds when b = 0.3 and μ = 0.85714 for the proposed protocols and other protocols.

**Fig. 8:**
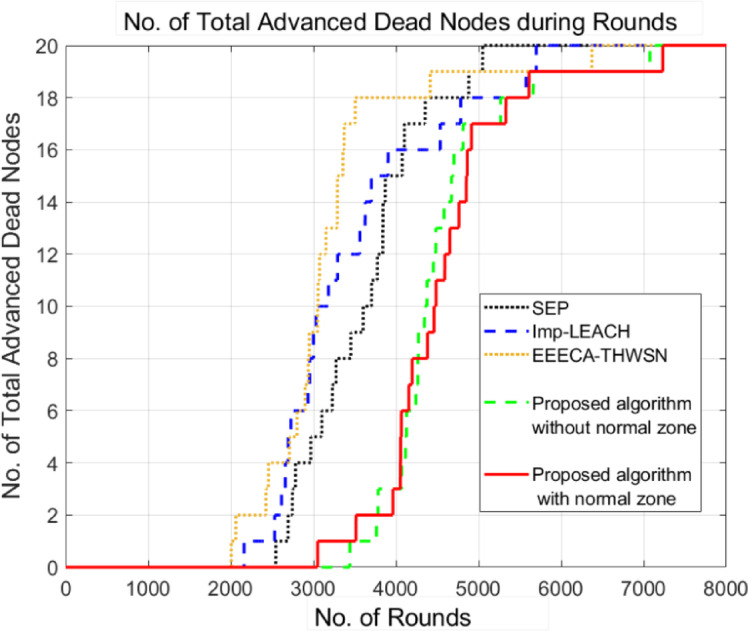
The total number of advanced dead nodes against number of rounds when m = 0.2 and α = 1.71428 for the proposed protocols with and without the normal zone and other protocols.

**Fig. 9:**
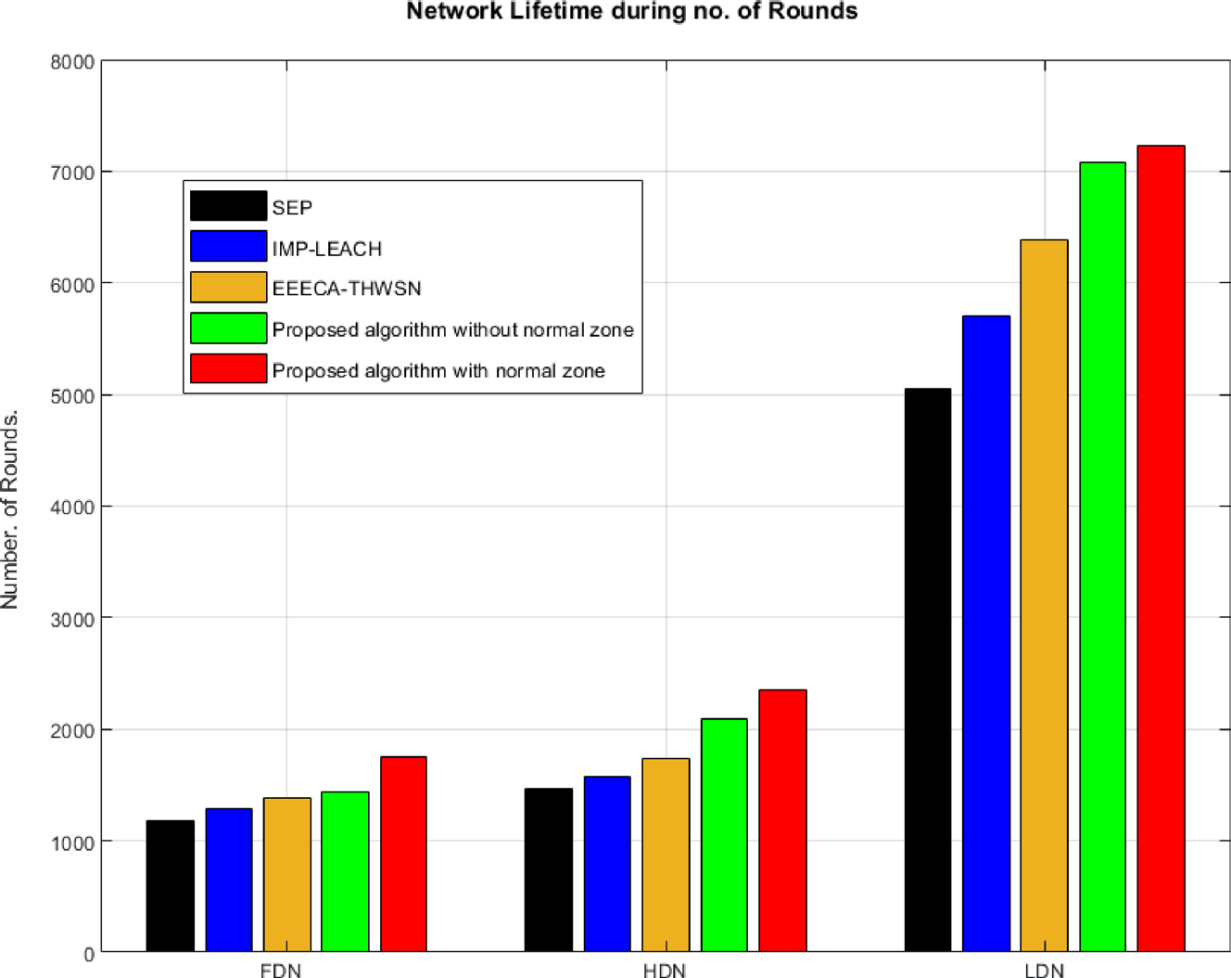
The network lifetime of the proposed protocols and other protocols during number of rounds, the proposed protocol with the normal zone is better in the first dead node (FDN), half dead node (HDN), and last dead node (LDN).

**Fig. 10:**
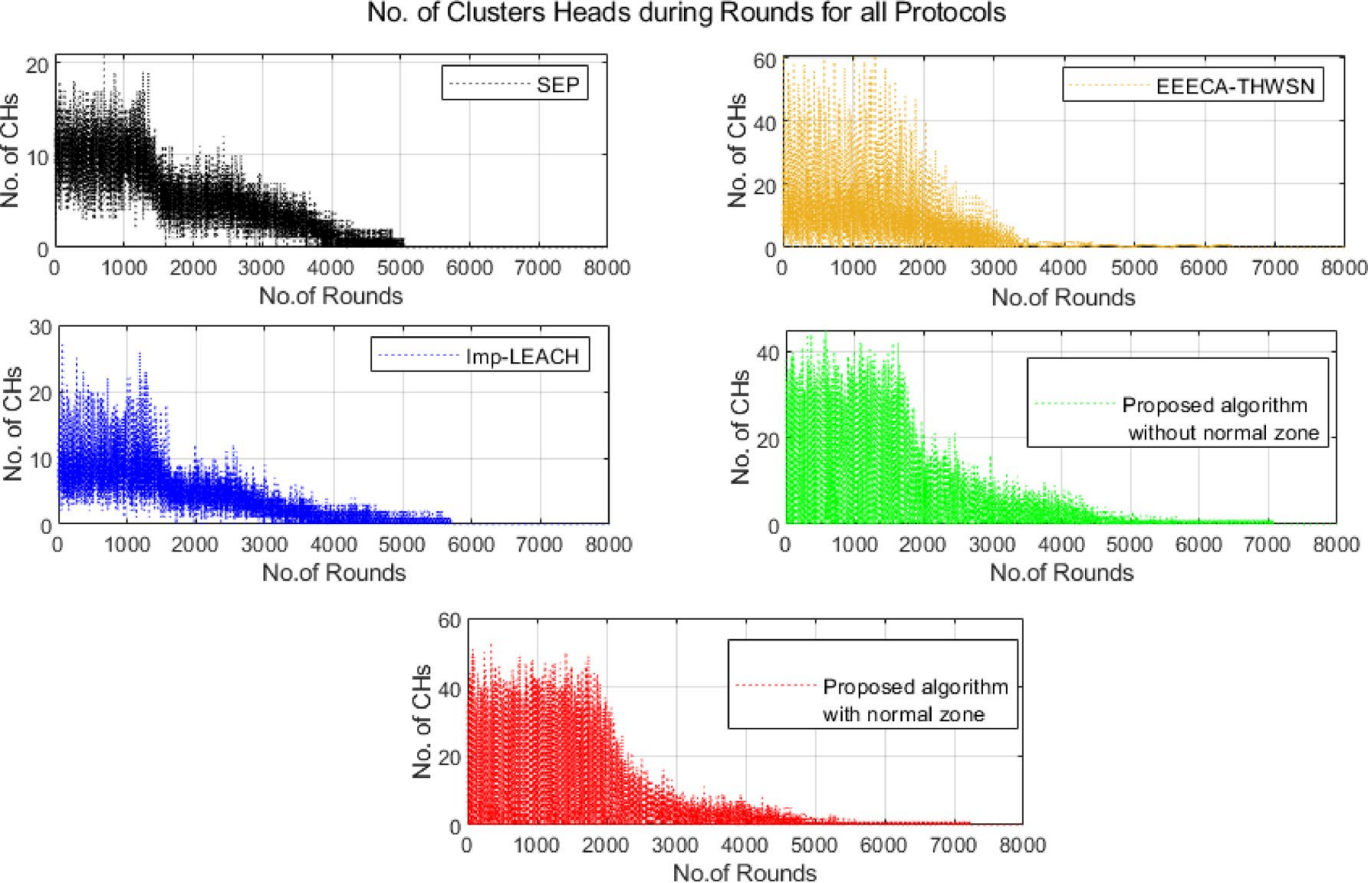
The number of cluster heads for all protocols versus number of rounds, the proposed protocol with the normal zone is better at CHs selection than other protocols.

**Fig. 11:**
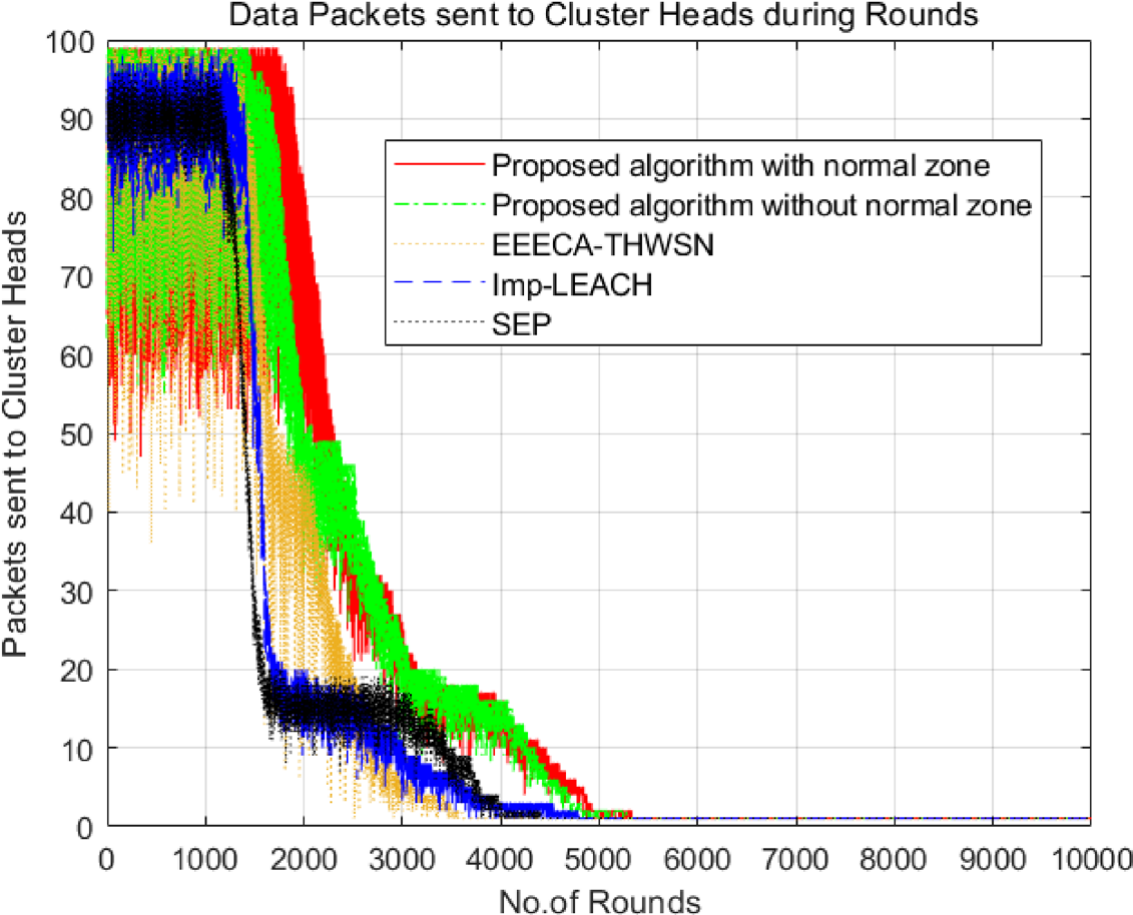
The data packets sent to cluster heads versus number of rounds, the highest data transmission rate to CHs of the proposed protocol with the normal zone.

**Fig. 12:**
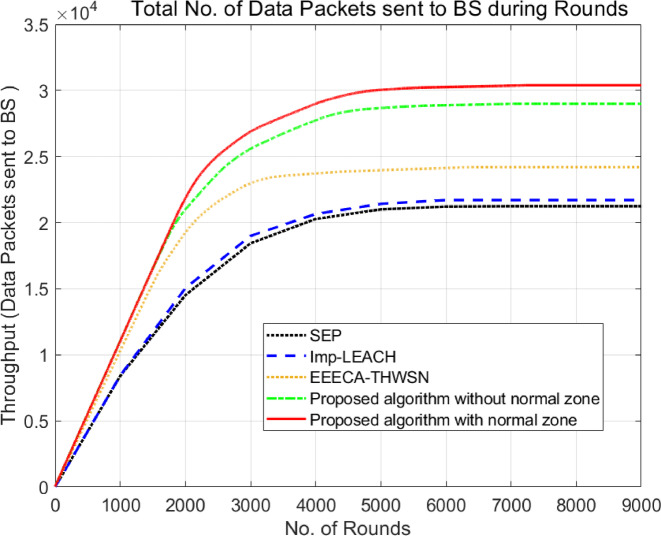
Total number of data packets sent to BS against number of rounds, the highest throughput to the BS for the proposed protocol with the normal zone against all protocols.

**Fig. 13:**
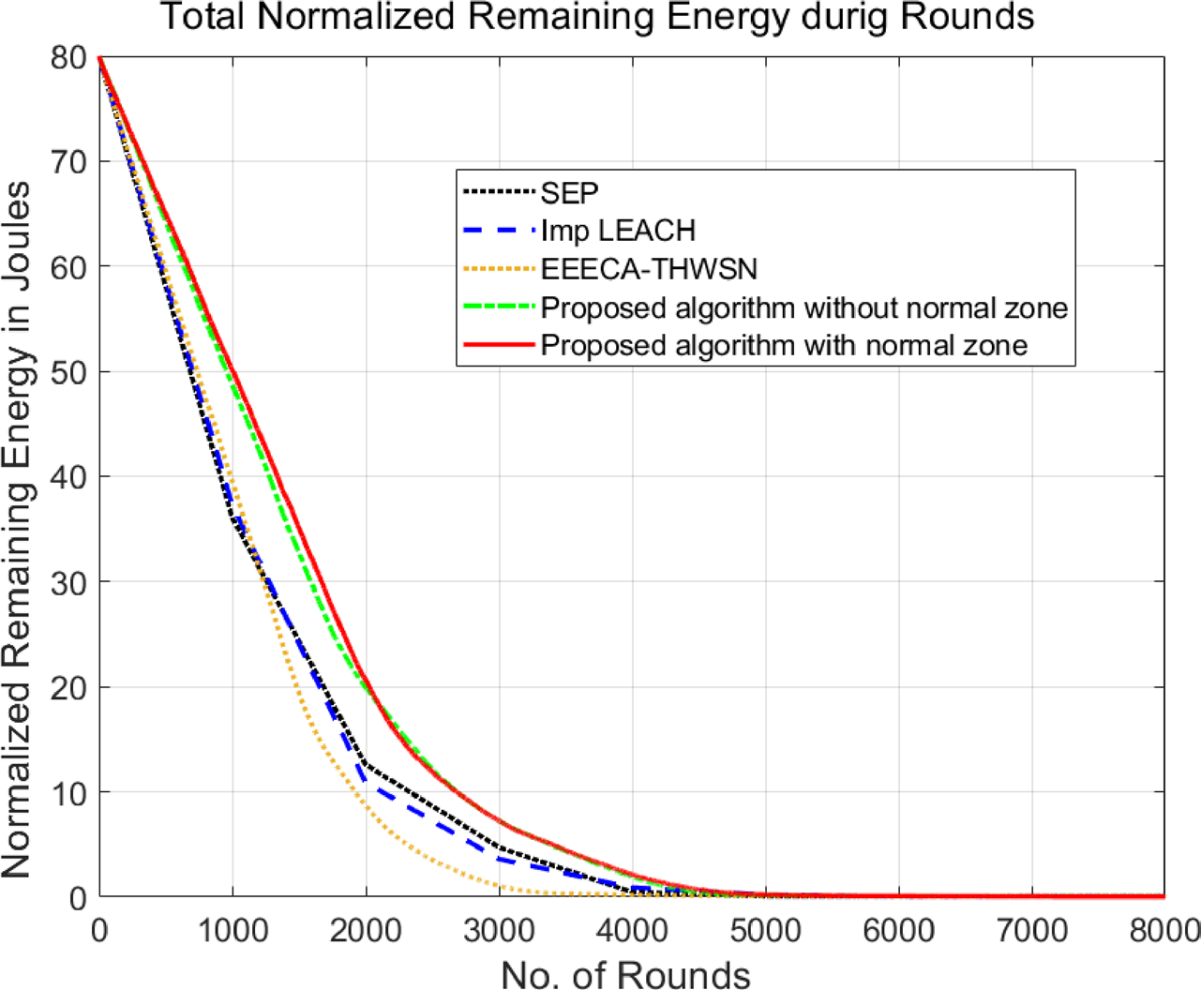
Total normalized remaining energy against the number of rounds when the energy used for all SNs in each protocol is consistently 80 Joules, the proposed protocol with the normal zone surpasses all others, maintaining higher energy levels up to 2200 rounds compared to others.

Figures 4, 5, 6, 7, 8, 9, 10, 11, 12, and 13 compare the proposed protocol with and without the normal zone against the previous protocols SEP, Imp-LEACH, and EEECA-THWSN. In each protocol, the total energy available for all SNs in the network is fixed at 80 Joules. The comparison is based on key performance metrics, including network lifetime, stability period (first dead node), the point at which half and all SNs have died, total dead nodes, throughput, and normalized remaining energy. Limiting the number of CHs significantly impacts performance, as a node is only elected as a CH if it has sufficient energy to sustain itself during the transmission period, and this influence is reflected in the performance metrics.

### Comparison of simulation results

Figures 4 and 5 compare the proposed protocol, both with and without the normal zone, against the previous protocols SEP, Imp-LEACH, and EEECA-THWSN in terms of the total number of dead nodes, network lifetime, and stability period across rounds, with all protocols using 80 Joules of energy for all SNs. The proposed protocol without the normal zone outperforms the other protocols, as the first dead node (FDN) appears at round 1433, representing the stability period. In contrast, the FDN in SEP, Imp-LEACH, and EEECA-THWSN occurs at rounds 1184, 1287, and 1378, respectively.

Network lifetime is measured from the start of operation until the last node dies (LND). In the proposed protocol without the normal zone, the last dead node (LDN) occurs at round 7074, while in SEP, Imp-LEACH, and EEECA-THWSN, the LDN occurs at rounds 5048, 5698, and 6379, respectively. This improvement is due to the reduction in cluster head (CH) extinction, as the protocol selects CHs based on the average energy of SNs and considers both energy and distance factors.

By incorporating the normal zone, the proposed protocol further enhances performance, especially in prolonging CH lifetime. This is achieved by addressing the long transmission distances faced by normal nodes deployed in Nrm-Zone1, which have lower energy compared to the advanced and intermediate nodes. As a result, the transmission distance between these nodes and the stationary BS is shorter. Consequently, in the proposed protocol with the normal zone, the FDN occurs at round 1755, and the LDN at round 7235, indicating even better performance. These results are detailed in Table 3.

**Table 3:** Comparison of proposed protocol (with and without normal zone) and other protocols.

Parameter	Protocols				
	SEP	Imp-LEACH	EEECA-THWSN	Proposed protocol without normal zone	Proposed protocol with normal zone
No. of heterogeneous levels	2	2	3	3	3
No. of zones	0	0	0	0	two
Stability period (first node death; FDN)	1184	1287	1378	1433	1755
Half node death (HND)	1460	1570	1735	2087	2352
Instability period (first to last dead)	3864	4411	5001	5641	5480
Network Lifetime (LDN) (from start operation to Last dead node)	5048	5698	6379	7074	7235
No. of Packets to BS (Throughput)	2.124 × 10^4^	2.171 × 10^4^	2.42 × 10^4^	2.9 × 10^4^	3.039 × 10^4^

Figures 6, 7, and 8 provide a comparison of the total number of dead normal, intermediate, and advanced nodes in the network, analyzed separately for the proposed protocol (with and without the normal zone) and the previous protocols. It is evident that the proposed protocol with the normal zone outperforms the other protocols in terms of the stability period (FDN) across all node types, FDN occurs at round 1755. The improvement is particularly notable for normal nodes, as their shorter transmission distance to the BS contributes to a longer stability period.

Figure 9 compares the network lifetime of the proposed protocol (with and without the normal zone) against SEP, Imp-LEACH, and EEECA-THWSN. This figure highlights the first dead node (FDN), half dead node (HDN), and last dead node (LDN). Clearly, the proposed protocol with the normal zone achieves better network lifetime results compared to the proposed protocol without the normal zone, as well as SEP, Imp-LEACH, and EEECA-THWSN, as illustrated in the figure and detailed in Table 3.

Figures 10 and 11 highlight the improvements in the heterogeneous clustering protocol for both the proposed protocol without the normal zone and the proposed protocol with the normal zone. Figure 10 shows the number of CHs during rounds for the proposed protocol in comparison to SEP, Imp-LEACH, and EEECA-THWSN. Since the proposed protocol uses three heterogeneous node types with different energy levels, advanced and intermediate nodes, when selected as CHs, remain active for longer due to their higher energy compared to normal nodes. Moreover, the proposed protocol enhances CH selection by considering both the energy levels of live SNs and their transmission distance to the BS.

The proposed protocol with the normal zone achieves further improvements by reducing the transmission distance from CHs of normal nodes to the BS. Consequently, Fig. 11 shows the data packets sent to CHs during rounds across all protocols, with the proposed protocol with the normal zone achieving the highest data transmission rate to CHs. This is a direct result of addressing long transmission distances and considering both energy and distance in node selection.

Based on the results presented earlier, Fig. 12 illustrates the throughput performance of the proposed protocol compared to other protocols by measuring the number of packets transmitted from CHs to the BS per round. The proposed protocol without the normal zone outperforms previous protocols by reducing CH extinction and improving CH selection based on distance and energy factors. Notably, the proposed protocol with the normal zone achieves even higher throughput to the BS than SEP, Imp-LEACH, and EEECA-THWSN, as it overcomes the challenges associated with long transmission distances for low-power nodes.

Since energy consumption is one of the most critical challenges in WSNs, Fig. 13 illustrates the total normalized remaining energy of the network over rounds, where the energy used for all SNs in each protocol is consistently 80 Joules. It is evident that the remaining energy of the proposed protocol without the normal zone remains higher than the other protocols from startup until over 5000 rounds. However, the proposed protocol with the normal zone surpasses all others, maintaining higher energy levels up to 2200 rounds compared to the proposed protocol without the normal zone, SEP, Imp-LEACH, and EEECA-THWSN, owing to its superior stability period. The summary of these results, based on Figs. 4, 5, 6, 7, 8, 9, 10, 11, and 12, is presented in Table 3, highlighting metrics such as the stability period (FDN), half node death (HDN), last node death (LDN), and throughput. It is clear from Table 3 that the proposed protocol with the normal zone outperforms the other protocols across all evaluation parameters.

The proposed protocols maintain low computational complexity by using simple, energy-efficient operations and reducing long-distance transmissions, especially for low-energy sensor nodes. Unlike SEP, which ignores transmission distance, our scheme adds minimal overhead by considering network heterogeneity. Compared to Imp-LEACH, which relies on complex multi-level clustering and ignores BS distance leading to rapid energy depletion of low-power CHs our approach remains lightweight and more efficient in both computation and communication.

The proposed protocol is designed to be lightweight and energy-efficient, which supports its scalability to larger or more dynamic wireless sensor network deployments. Its low computational and communication overhead makes it suitable for implementation on real-world, resource-constrained devices such as low-power sensor nodes. Potential application areas include smart agriculture, environmental monitoring, and industrial IoT scenarios.

## Conclusion

In WSNs, energy-efficient utilization and extending the network’s lifetime are critical challenges when designing routing protocols. This paper introduced enhanced cluster-based proactive routing protocols for three-tier energy-heterogeneity WSNs, presenting two variations: the proposed protocol without normal zone and the Proposed Protocol with normal zone. Both protocols consider energy and distance factors of SNs in relation to the BS for CH selection, giving nodes with higher energy and closer proximity to the BS a higher likelihood of being elected as CHs. Additionally, the protocols prevent the creation of unnecessary CHs for SNs with low remaining energy by incorporating weighted probabilities based on the average energy of live SNs, thereby reducing wasted energy.

Furthermore, the proposed protocol with normal zone addresses the challenge of long transmission distances for low-power nodes by distributing these nodes in a designated Nrm-Zone1, reducing transmission distances and improving energy efficiency. Simulation results demonstrate that the proposed protocol with normal none significantly outperforms the proposed protocol without normal zone, as well as the SEP, IMP-LEACH, and EEECA-THWSN protocols in terms of network lifetime, stability period (first dead node), half and last node deaths, throughput, and normalized remaining energy.

Building upon the promising results of the enhanced cluster-based proactive routing protocols, future work will focus on further optimizing energy efficiency in more dynamic environments. One area of exploration is the incorporation of mobility within the sensor nodes and BS, where the network must adapt to changing topologies. Additionally, investigating machine learning algorithms for adaptive CH selection based on real-time energy consumption and environmental conditions could further enhance the network’s resilience and performance.

## Data Availability

All simulation results were done on MATLAB, and can be provided by the corresponding author on reasonable request.
